# MicroRNA-mediated regulation of p21 and TASK1 cellular restriction factors enhances HIV-1 infection

**DOI:** 10.1242/jcs.167817

**Published:** 2015-04-15

**Authors:** Luba Farberov, Eytan Herzig, Shira Modai, Ofer Isakov, Amnon Hizi, Noam Shomron

**Affiliations:** Faculty of Medicine, Tel Aviv University, Tel Aviv 69978, Israel

**Keywords:** Human immunodeficiency virus-type 1, miRNA, Cellular restriction factor, Cyclin-dependent kinase inhibitor, TASK1

## Abstract

MicroRNAs (miRNAs) are short non-coding RNAs that play a central role in the regulation of gene expression by binding to target mRNAs. Several studies have revealed alterations in cellular miRNA profiles following HIV-1 infection, mostly for miRNAs involved in inhibiting viral infection. These miRNA expression modifications might also serve to block the innate HIV-1 inhibition mechanism. As a result, it is expected that during HIV-1 infection miRNAs target genes that hinder or prevent the progression of the HIV-1 replication cycle. One of the major sets of genes known to inhibit the progression of HIV-1 infection are cellular restriction factors. In this study, we identified a direct miRNA target gene that modulates viral spread in T-lymphocytes and HeLa-CCR5 cell lines. Following infection, let-7c, miR-34a or miR-124a were upregulated, and they targeted and downregulated p21 and TASK1 (also known as CDKN1A and KCNK3, respectively) cellular proteins. This eventually led to increased virion release and higher copy number of viral genome transcripts in infected cells. Conversely, by downregulating these miRNAs, we could suppress viral replication and spread. Our data suggest that HIV-1 exploits the host miRNA cellular systems in order to block the innate inhibition mechanism, allowing a more efficient infection process.

## INTRODUCTION

Human immunodeficiency virus type 1 (HIV-1), the causative agent of acquired immune deficiency syndrome (AIDS), was first described in 1983 (Barré-Sinoussi et al., 1983; Gallo et al., 1983). According to the latest 2013 UNAIDS report, AIDS has already caused the death of >30 million patients, and 35 million people are estimated to be living with HIV worldwide (http://www.unaids.org). The viral infection process includes interaction of the viral envelope protein (gp120) with the host-cell CD4 receptor, resulting in viral entry, reverse transcription to generate proviral cDNA, integration of this cDNA into the human genome, RNA transcription, viral protein synthesis and virion assembly, culminating in budding (Strebel et al., 2009; Liu et al., 2011). Although there are already many effective drugs capable of controlling viral replication and disease progression (Swaminathan, S. et al., 2012), ongoing attempts to develop a useful HIV-1 vaccine are unlikely to be successful in the near future, given that HIV-1 has proven to be capable of rapidly developing resistance to therapy, evading the immune response, altering cellular immune function and inhibiting apoptosis in infected cells (Weiss, 1993; Klase et al., 2009; Strebel, 2013). A better understanding of innate inhibition mechanisms of host and HIV can potentially promote HIV-1 therapeutics (Santa-Marta et al., 2013).

Cellular restriction factors are host proteins that hinder or prevent the progression of different steps in the HIV-1 replication cycle (Sheehy et al., 2002; Harris et al., 2012; Strebel, 2013; Rehwinkel, 2014). This innate inhibition mechanism includes several proteins, such as APOBEC3G, tetherin (also known as BST2), cyclophilin A (also known as PPIA), Trim5α, TRIM28, p21 (also known as CDKN1A), SAMHD1, PAF1, UBP (also known as SGTA) and TASK-1 (also known as KCNK3). In this communication, we focused on p21 and TASK, because our screens revealed them as potential targets for miRNAs following HIV-1 infection. The p21 protein is a cyclin-dependent kinase inhibitor that negatively regulates the G1-S transition. This factor can independently block HIV-1 reverse transcription and mRNA transcription, by hindering the essential transcriptional elongation factor CDK9 (Yu and Lichterfeld, 2011). TASK1 is a human potassium channel protein, which was found to be able to obstruct the ability of the viral protein Vpu to enhance viral particle release (Hsu et al., 2004). However, the mechanisms by which these restriction factors function is not completely understood.

The microRNA (miRNA) pathway is one of the mechanisms that regulate the immune response to pathogenic infections. miRNAs are small non-coding RNAs (ncRNAs) of ∼22 nucleotides that guide post-transcriptional repression of protein-coding genes by base-pairing with the 3′ untranslated region (3′UTR) of a target mRNA. The 5′-positioned 2–8 nucleotides of the miRNA (also known as the ‘seed’ region) bind to the mRNA sequence to exert their functional regulation (Kim et al., 2009; Klase et al., 2009). Through this short complementary base-pairing, one miRNA can potentially regulate the expression of hundreds of different independent mRNAs (Grimson et al., 2007; Shomron, 2009; Strebel et al., 2009). Biogenesis of miRNAs includes transcription by RNA polymerase II, processing of the generated double-stranded RNA (dsRNA) by Drosha and Dicer to a mature miRNA, which is loaded onto the RNA-induced silencing complex (RISC). The miRNA–RISC complex can lead to degradation of the target mRNA or to inhibition of translation initiation (Bushati and Cohen, 2007; Shomron, 2009; Esteller, 2011). Currently, there are several hundreds of reported human miRNAs (http://www.mirbase.org) that are predicted to control >60% of protein-coding genes (Kozomara and Griffiths-Jones, 2014). Many studies have shown alterations in cellular miRNAs profiles following HIV-1 infection (Yeung et al., 2005; Triboulet et al., 2007; Houzet et al., 2008). Most of them reveal that the shift in miRNA expression results in viral replication inhibition (Strebel et al., 2009; Swaminathan et al., 2012; Swaminathan et al., 2014) by directly targeting HIV-1 (Huang et al., 2007; Ahluwalia et al., 2008; Nathans et al., 2009; Wang et al., 2009; Chen et al., 2014) or by modulating the expression of HIV dependency factors (HDFs) (Triboulet et al., 2007; Sung and Rice, 2009; Shen et al., 2012; Swaminathan et al., 2012). Only a few publications have described host miRNAs that enhance HIV-1 infection (Zhang et al., 2012b; Zhang et al., 2012a; Chiang et al., 2013; Orecchini et al., 2014). No study to date looked into the possible interactions between miRNAs and restriction factors in the process of HIV-1 infection (Swaminathan et al., 2014). Our work directly links HIV-1 infection, miRNAs and the levels of cellular restriction factors. Specifically, an increased amount of host let-7c downregulates p21, and miR-34a and/or miR-124a regulates TASK1. The inhibition of these proteins enhances viral replication, pathogenesis and survival of the virus. Our study extends the knowledge on the mechanism of HIV-1 infection and regulation.

## RESULTS

In order to understand the changes in miRNA expression following HIV-1 infection and how it regulates restriction factors and the course of viral infection, we profiled the miRNA expression prior to and post infection (8 days) in two lymphocyte cell lines (Sup-T1 and T1). Using the deep-sequencing method, we detected 151 miRNAs that were differently expressed with greater than a twofold change. In the Sup-T1 cell line, 25 miRNAs were downregulated and 126 miRNAs were upregulated, whereas in the T1 cell line, 36 miRNAs were upregulated and 54 miRNAs were downregulated by twofold or greater (supplementary material Table S1A–D). Using TaqMan low-density array (TLDA) on the same Sup-T1 samples, we identified 60 downregulated miRNAs and 72 upregulated miRNAs with greater than a twofold change (supplementary material Table S2A,B). Various groups have studied the miRNA profile after HIV-1 infection; for example, Chang et al. used deep sequencing to identify differently expressed miRNAs at 5, 12 and 24 hours after HIV-1 infection in the sup-T1 cell-line, and found 21 miRNAs that were differently expressed at 24 hours post infection, with the majority being upregulated. Ten miRNAs accounted for >70% of all miRNA-mapped reads; among the most highly expressed miRNAs were several members of the let-7 family (Chang et al., 2013). Pacifici et al. identified 66 miRNAs that were found to be differentially regulated in HIV-positive compared with HIV-negative groups in cerebrospinal fluid (CSF). Upon additional evaluation of miRNA profiles in archived brain tissues, 121 differentially regulated miRNAs were observed. In both experiments, the authors found an overall downregulation in miRNA expression (Pacifici et al., 2013). Witwer et al. used the NanoString, TLDA and quantitative (q)PCR methods in order to profile miRNA expression in peripheral blood mononuclear cells (PBMCs) and found several differently expressed miRNAs, most of which were downregulated in viremic samples (Witwer et al., 2012). Yeung et al. profiled miRNAs differently expressed in mock-transfected HeLa cells versus HeLa cells transfected with an infectious HIV-1 molecular clone, pNL4-3. The authors found that, although the majority of miRNAs remained unchanged, ∼43% of the miRNAs were more than twofold downregulated (Yeung et al., 2005). All of these previous studies show the inconsistency in miRNA expression patterns following viral infection.

### miRNA expression profiling following HIV-1 infection

**Fig. 1.**
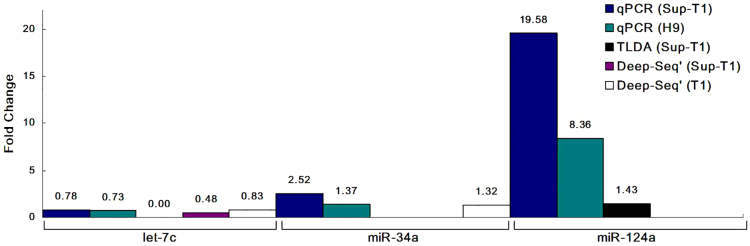
**Expression of let-7c, miR-34a and miR-124a at 8 days following HIV-1 infection.Expression of let-7c, miR-34a and miR-124a at 8 days following HIV-1 infection.** Normalized fold change results are presented for deep sequencing (Sup-T1 and T1), TLDA (Sup-T1) and qPCR (Sup-T1 and H9).

Most literature addressing changes in miRNA expression following HIV-1 infection shows an inhibiting effect on viral spread (Strebel et al., 2009; Swaminathan et al., 2014). Given that an estimated 50% of all cellular genes are regulated by miRNAs (Friedman et al., 2009), it seemed only reasonable that some HIV-1 restriction factors would also be regulated by miRNAs. The anti-correlative relationship between miRNAs and their target genes suggests one pattern in which miRNAs increase following viral infection, resulting in decreased expression of their restriction factor target genes, which would overall be beneficial for viral replication. We therefore sought to determine the direct miRNA–gene-function relationship. For this purpose, we performed miRNA expression profiling in T cell lines (Sup-T1, H9 and T1) prior to and post (8 days) HIV-1 infection, using deep sequencing and real-time PCR array (TLDA; see Materials and Methods). The overall results from multiple platforms showed that, following viral infection, let-7c was downregulated, whereas hsa-miR-34a and hsa-miR-124a were upregulated (Fig. 1). These results correspond to previous findings where let-7c was upregulated on the first day after viral infection and downregulated at later time points. Modulation of miRNA expression has been observed, with different patterns in several cell lines and at different stages of HIV-1 infection. Zhang et al. have shown that miR-34a is significantly upregulated following viral infection (Zhang et al., 2012b), which was also found by Mohammadi et al., who also reported upregulation of let-7b and miR-124 (Mohammadi et al., 2013). Chang et al. observed significant upregulation of let-7c family members in Sup-T1 cells at 24 hours post infection with HIV-1 (Chang et al., 2013). Yeung et al. have reported upregulation of let-7c in HeLa cells transfected with the infectious HIV-1 molecular clone pNL4-3 (Yeung et al., 2005). Pacifici et al. have shown that let-7c is downregulated in the CSF and brains of patients (Pacifici et al., 2013). The same miRNA expression profile was observed following HIV-2 (ROD strain) infection of an H9 lymphocyte cell line; at 24 hours after viral infection, all three miRNAs were upregulated, whereas at 48 hours post HIV-2 infection, miR-34a and miR-124a remained overexpressed and let-7c was downregulated (supplementary material Fig. S1). miRWalk, which uses various algorithms that predict potential binding sites of miRNAs on target genes (Dweep et al., 2011), indicated a high probability of let-7c for targeting CDKN1A, and also showed that miR-34a and miR-124a might regulate TASK1. This corresponded with the prediction by Chang et al. that let-7b might target CDKN1A mRNA (Chang et al., 2013). Changes in miRNA expression profiles, as shown in Fig. 1, suggest that let-7c, miR-34a and miR-124a play a role in gene regulatory networks following HIV-1 infection.

### miRNAs target p21 and TASK1

To prove the regulation of a target gene by miRNA, one expects that the following conditions apply: (1) anti-correlated expression between the miRNA and the mRNA levels upon miRNA manipulation (increasing or decreasing levels of the miRNA); (2) protein downregulation as observed by a reporter system (e.g. luciferase assays) and its reversibility following mutation in the miRNA binding site.

**Fig. 2.**
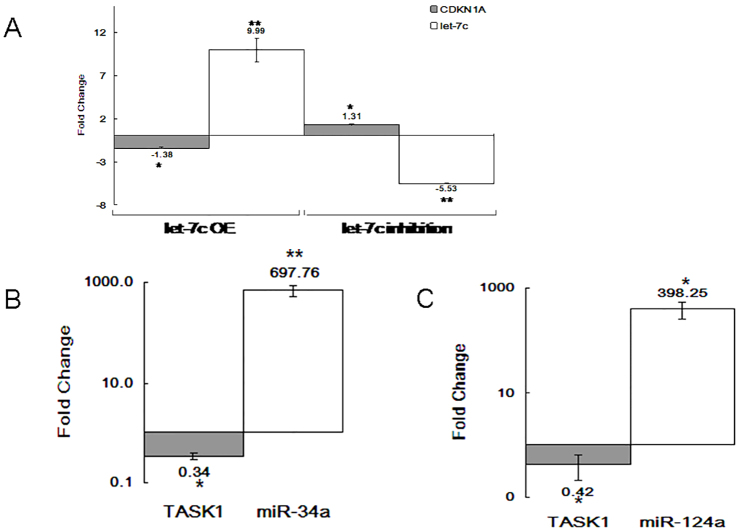
**Target genes affected by miRNA levels.Target genes affected by miRNA levels.** Real-time PCR analysis of miRNA and gene expression following 48 hours of miRNA overexpression (OE) or inhibition in the Jurkat cell line. The data show the normalized fold change of (A) let-7c and CDKN1A, and the normalized logarithmic fold change of (B) miR-34a and TASK1, and (C) miR-124a and TASK1, relative to the expression of the control plasmid. Data show the mean±s.e.m. (*n* = 3); *P<0.05, **P<0.005 (paired Student's *t*-test).

**Fig. 3.**
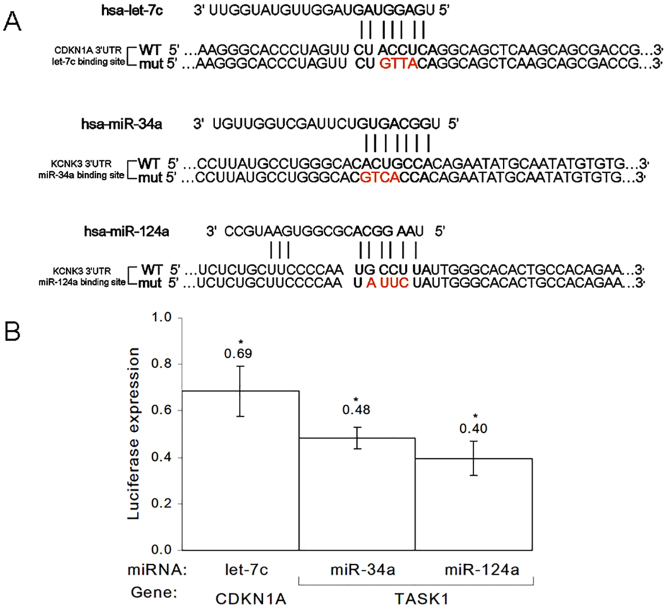
**Luciferase reporter activity indicates direct binding and regulation of miRNAs.Luciferase reporter activity indicates direct binding and regulation of miRNAs.** (A) Sequences of *Renilla* or firefly luciferase under regulation of CDKN1A or KCNK3 3′UTRs that were used for transient reporter assay experiments. Wild-type (WT) and mutant (mut) alleles for each of the three miRNA-binding sites are presented. The miRNA seed region and complimentary 3′UTR sequence are marked in bold. Mutagenized nucleotides are in red and bold. (B) Luciferase activity at 48 hours after co-transfection of different miRNA combinations with *Renilla* and firefly luciferase constructs under regulation of the CDKN1A or TASK1 3′UTR. The data presented are the relative levels of *Renilla* luciferase expression standardized to those of *firefly* luciferase. Data show the mean±s.e.m. (*n* = 3); *P<0.05 (paired Student's *t*-test).

**Fig. 4.**
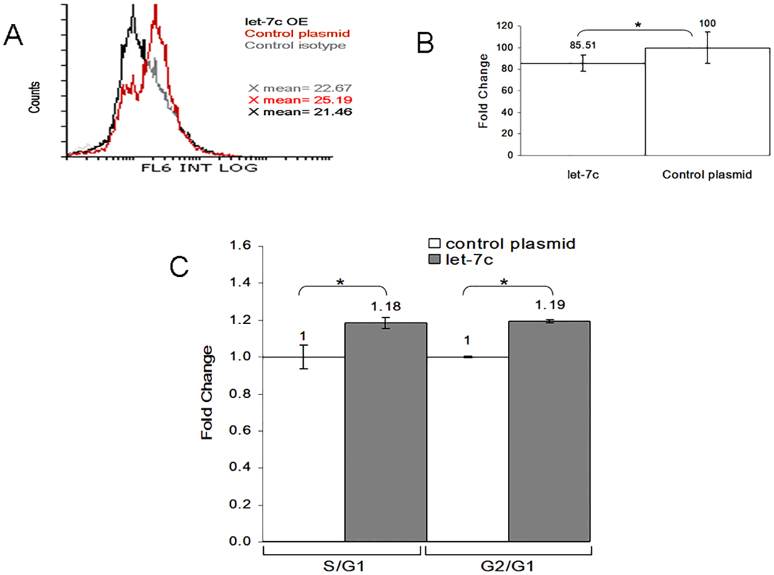
**The p21 protein is regulated by let-7.The p21 protein is regulated by let-7.** Flow cytometric analysis of p21 expression following 72 hours of let-7c overexpression (OE) in the Jurkat cell line, (A) sample result and (B) quantification of three experiments. (C) Cell cycle is affected by let-7 and p21 levels. At 4 days following let-7c overexpression in the Jurkat cell line, anti FITC-BrdU and 7-AAD were used in order to determine the proportion of cells in each cell cycle phase. Analysis was performed using Flowing Software 2 (Cell Imaging Core, Turku Centre for Biotechnology, Finland). The S:G1 and G2:G1 ratios in cells treated with let-7c or control are presented. Data in B,C show the mean±s.e.m. (*n* = 3); *P<0.05 (paired Student's *t*-test).

In order to validate the predicted functional interaction between let-7c and CDKN1A or between miR-34a or -124a and TASK1, we set out to quantify the levels of the relevant mRNA (by RT-PCR, Fig. 2A–C) and protein (by flow cytometry and western blotting, Fig. 4B; supplementary material Fig. S2). In addition, we also carried out a direct binding assay (luciferase) in order to establish the interaction between the miRNAs and their gene targets (Fig. 3A,B). Moreover, we were interested in determining whether there was a change in cell cycle and proliferation after let-7c-mediated p21 downregulation (by using flow cytometry, Fig. 4C).

Three miRNA vectors (miRVec, see Materials and Methods) expressing let-7c, miR-34a or miR-124a miRNA, were transfected into the Jurkat cell line for 48 hours. Real-time PCR reactions carried out on each transfectant revealed that overexpression of the let-7c miRNA led to a 1.38-fold decrease in CDKN1A mRNA expression (Fig. 2A), and overexpression of miR-34a and miR-124a reduced the levels of TASK1 mRNA by 66% and 58%, respectively (Fig. 2B,C). Additionally, an inverse expression pattern was observed after transfecting a synthetic let-7c antisense (AntagoMiR, see Materials and Methods) RNA sequence into the Jurkat cell line for 48 hours (Fig. 2A). An empty plasmid (miRVec) or a scrambled RNA sequence were used as controls for baseline expression, while GAPDH and miR-181a served as endogenous gene controls (see Materials and Methods).

For the purpose of establishing the direct interaction and binding of let-7c to CDKN1A and that of miR-34a or miR-124a to TASK1, we cloned a portion of the 3′UTR of each respective target gene into a luciferase reporter assay plasmid and co-transfected it, along with a miRNA vector plasmid, into the HeLa-CCR5 cell line (see Materials and Methods). After 48 hours of miRNA overexpression, *Renilla* luciferase and firefly luciferase expression was measured. In Fig. 3B, we show that the transfection of plasmids containing the wild-type 3′UTR of CDKN1A or TASK1 resulted in relatively lower luciferase activity as compared with that observed following transfection of plasmids containing the mutant 3′ UTR (luciferase activity was reduced to 0.69, 0.48 and 0.40 relative to mutant levels for the CDKN1A-let-7c, TASK1-miR-34a and TASK1-miR-124a pairings, respectively).

Following 72 hours of miRNA overexpression in Jurkat cells, the intracellular protein levels were measured by western blotting and flow cytometry (normalized to an empty control plasmid). Flow cytometric analysis showed a 14.5% downregulation of p21 in miRNA-overexpressing Jurkat cells (Fig. 4B) relative to controls, whereas western blotting demonstrated a 17% reduction in H9 cells (supplementary material Fig. S2).

Because p21 is a negative regulator of the G1-S transition in the cell cycle, we wanted to determine the effect of p21 downregulation on cell proliferation and cell cycle. At 4 days after let-7c overexpression in the Jurkat cell line, cell cycle and cell proliferation were measured. BrdU and 7-AAD staining indicated an 18% increase in the S:G1 ratio and a 19% rise in the G2:G1 ratio (Fig. 4C).

In conclusion, we observed a direct downregulation of p21 expression as a result of let-7c upregulation, with subsequent effects on cell cycle and cell proliferation. Furthermore, increased expression of miR-34a and miR-124a reduced TASK1 mRNA levels. This indicates that these miRNAs directly regulate their target restriction factors.

### HIV-1 infectivity is modified following miRNA manipulation

**Fig. 5.**
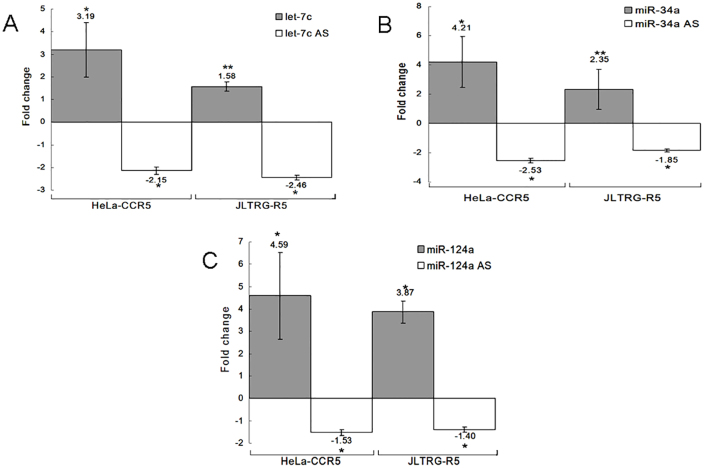
**Functional consequences of miRNA levels on viral replication and virion release to the supernatant.Functional consequences of miRNA levels on viral replication and virion release to the supernatant.** Real-time PCR of relative viral LTR expression in the supernatant at 24 hours (A) or 5 days (B,C) following HIV-1 infection of HeLa-CCR5 and JLTRG-R5 cell lines, and overexpression or inhibition of (A) let-7c, (B) miR-34a or (C) miR-124a. AS, antisense. Data show the mean±s.e.m. (*n* = 3); *P<0.05, **P<0.005 (paired Student's *t*-test).

**Fig. 6.**
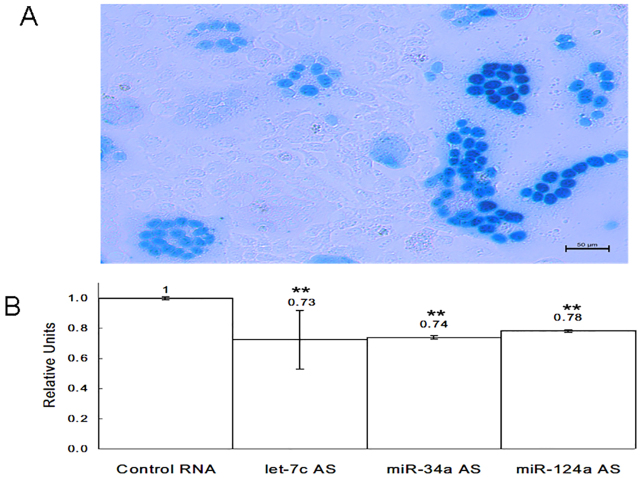
**Overexpression of miRNA inhibitors reduces HIV-1 viral replication.**(A) Representative picture of X-gal-stained HeLa-CCR5 cells infected with HIV-1. The photograph was taken using light microscopy. (B) The data represent the number of relative units (blue cells) counted in AntagoMiR-overexpressing wells relative to control sequence wells [miR antisense (AS):scrambled sequence]. Data show the mean±s.e.m. (*n* = 3); **P<0.005 (paired Student's *t*-test).

Following our observation of restriction factor downregulation by the three selected miRNAs, we were compelled to see how their expression profile affects HIV-1 and its virulence process. In order to address this issue, we infected HeLa-CCR5 and JLTRG-R5 cells at 48 hours after miRNA overexpression or inhibition. Supernatant samples were collected and analyzed by RT-PCR from both cell lines at 24 hours post infection for let-7c, and 5 days post infection for miR-34a and miR-124a. A direct correlation between the level of miRNAs and viral transcripts was observed for all three miRNAs in all experiments. miRNA overexpression resulted in an increase in virion release, and miRNA inhibition led to a decline in viral replication (Fig. 5A–C). Next, we performed a multinucleate activation of galactosidase indicator (MAGI) infection assay in order to test how miRNA inhibition affected viral replication. The HeLa-CCR5 cells were infected with HIV-1 at 48 hours post transfection with let-7c, miR-34a or miR-124a inhibitors. The results showed a reduction in the number of infected cells in the experimental wells compared with the control ones. At 24 hours post infection, let-7c inhibition lowered the relative virion release (the number of blue cells counted) by 27%; whereas, at 5 days post infection, transfection of cells with miR-34a and miR-124a antisense oligonucleotides lowered the number of infected cells by 26% and 22%, respectively (Fig. 6A,B).

**Fig. 7.**
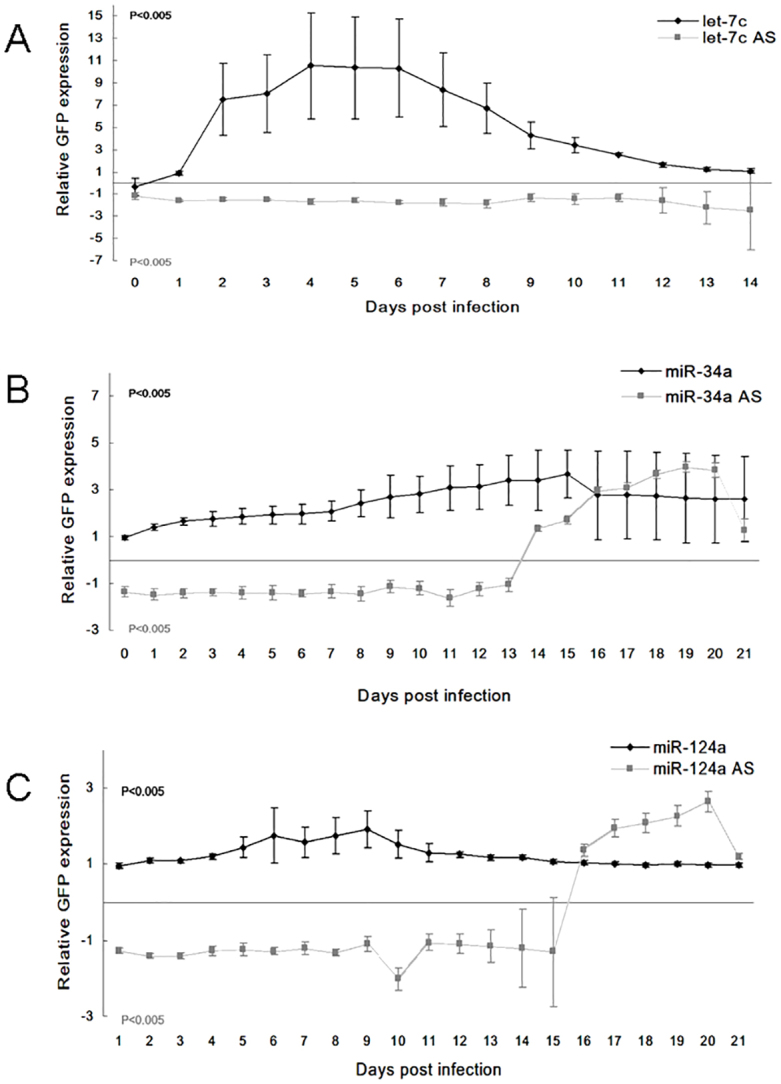
**Long-term functional consequences of miRNA expression on viral replication and presence in the cells.Long-term functional consequences of miRNA expression on viral replication and presence in the cells.** Viral LTR expression in the JLTRG-R5 cell line up to 14 or 21 days. The data show the effects of miRNA overexpression relative to control plasmid (miR:empty vector, black line) and miRNA inhibition relative to control sequence [miR antisense (AS):scrambled sequence, gray line] of (A) let-7c, (B) miR-34a and (C) miR-124a. Data show the mean±s.e.m. (*n* = 3); *P*<0.005 (paired Student's *t*-test for miR treatment versus control).

In order to study the long-term effect of miRNA overexpression or inhibition, we infected the JLTRG-R5 cell line with HIV-1 at 48 hours post miRNA treatment. The relative treatment and control GFP expression was measured, showing a direct correlation between the amount of miRNA and virus in all experiments. The quantified long terminal repeat (LTR) reads were indicative of HIV-1 replication. We obtained higher LTR reads for 2 weeks following let-7c overexpression, in contrast to the overexpression of miRNA antisense oligonucleotides, which resulted in a lower viral production (Fig. 7A). The same pattern was seen for a period of 21 days following miR-34a and miR-124a overexpression, and for 15 days with miRNA inhibition (Fig. 7B,C).

In conclusion, the miRNA expression profile can either strengthen (miRNA overexpression) or weaken (miRNA inhibition) the infection process and have a profound impact on viral replication both in the supernatant and in the infected cells. We found a direct relationship between the levels of miRNA and virus for three different cellular miRNAs, and an inverse correlation between miRNA and restriction factor expression.

## DISCUSSION

HIV-1 is the causative agent of AIDS. According to the 2013 UNAIDS report, >30 million people have died from AIDS, with 1.6 million deaths in the past year alone, and 2.3 million newly infected people worldwide. Owing to the ability of the virus to evade the immune system (Weiss, 1993), no vaccine has proven effective to date. As it stands, at over 30 years after its discovery, AIDS still remains an incurable disease.

Cellular restriction factors, a part of the innate human cellular processes, are able to delay viral spread. It is believed that they might serve as a potential therapeutic avenue. Owing to their crucial role in the obstruction of the virus replication cycle, these restriction factors are believed to act as potential targets for miRNAs in the early stages of the infection process. Two restriction factors, p21 and TASK, were examined in this paper.

miRNAs, short non-coding RNAs of ∼22 nucleotides, guide the post-transcriptional repression of protein-coding genes. Several studies have previously shown alterations in cellular miRNA profiles following HIV-1 infection, generally resulting in viral inhibition (Chiang and Rice, 2012; Swaminathan et al., 2014). For example, Huang et al. (Huang et al., 2007) found that cellular miRNAs contribute to HIV-1 latency in resting T-lymphocytes, and Ahluwalia et al. (Ahluwalia et al., 2008) and Nathans et al. (Nathans et al., 2009) proved that human miR-29a interferes with viral Nef protein expression and HIV-1 replication. Both Wang et al. (Wang et al., 2009) and Huang et al. (Huang et al., 2007) demonstrated how increased expression of cellular miR-28, miR125b, miR150, miR223 or miR382 inhibited HIV-1 replication in macrophages through sequence complementarity and binding to the viral mRNA. Sung et al. (Sung and Rice, 2009) indicated an anti-viral function of human miR-198, which targets cyclin T1 expression that is required for Tat transactivation. Similarly, Triboulet et al. (Triboulet et al., 2007) showed that overexpression of miR-17-5p and miR-20a resulted in reduced mRNA and protein levels of PCAF, an important cofactor for Tat in HIV-1 gene expression. Shen et al. (Shen et al., 2012) reported that inhibition of miR-15a, miR15b, miR16, miR20a, miR93 or miR106b in monocytes enhanced expression levels of Pur-α and led to increased HIV-1 infection. Finally, Swaminathan et al. (Swaminathan et al., 2012) reported that enhanced miR-155 expression levels correlated with decreased HIV-1 infectivity. Recently, Chen et al. showed that miRNA binding to the HIV-1 Gag protein inhibits Gag assembly and virus production (Chen et al., 2014).

In contrast to all of these findings, Chiang et al. (Chiang et al., 2013) showed for the first time in 2013 that miR-132 upregulation enhances HIV-1 replication. Recently, Orecchini et al. (Orecchini et al., 2014) suggested that HIV-1 reduces CD4 expression in infected cells by upregulating miR-222, resulting in the direct repression of the receptor. Two additional papers by Zhang et al. (Zhang et al., 2012b; Zhang et al., 2012a), found that miR-217 and miR-34a were significantly upregulated upon Tat exposure and enhanced HIV-1 Tat-mediated transactivation. This finding led us to further investigate whether HIV-1 infection can upregulate miRNAs that target restriction factors while interfering with the ability of the innate immune system to eradicate viral spread and promote the infection process.

We addressed this question by revealing the short- and long-term (up to 21 days post infection) effect of human let-7c, miR-34a and miR-124a overexpression or inhibition on the viral replication, spread and presence in the JLTRG-R5 and HeLa-CCR5 cells and the virion release to the supernatant by these cells.

Our work revealed three human miRNAs for which expression was upregulated following HIV-1 infection – let-7c, miR-34a and miR-124a. We found that let-7c targets CDKN1A, at the RNA and protein (p21) levels, whereas miR-34a and miR-124a regulate TASK1 mRNA expression. In addition, we observed that p21 downregulation resulted in enhanced cell cycle and cell proliferation. Using infection assays in the unique HeLa-CCR5 and JLTRG-R5 cell lines, we observed that overexpression of human let-7c, miR-34a and miR-124a miRNAs resulted in enhanced viral replication and spread both in the supernatant and in the cells. Inhibition of these three miRNAs resulted in a lower viral replication rate and a reduction in total virion production when compared to the control samples.

**Fig. 8.**
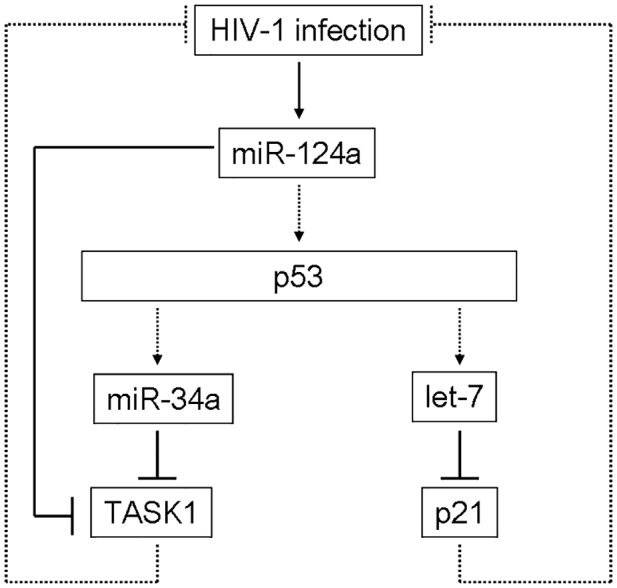
**Suggested p53 pathway associated with HIV-1.Suggested p53 pathway associated with HIV-1.** HIV-1 infection upregulates miR-124a expression (solid arrow), which will increase p53 protein expression (Shi et al., 2013). Human miRNAs let-7c and miR-34a are direct targets of p53 (Tarasov et al., 2007; Boominathan, 2010) and are upregulated as well (dashed arrows). The let-7c, miR-34a and miR-124a upregulation decreases p21 and TASK1 expression (solid lines). Downregulation of restriction factors strengthens the infection process (dashed lines).

miRNAs have a profound impact on the mechanistic comprehension of HIV-1 infection. High miRNA expression following HIV-1 infection targets inhibitory cellular factors, strengthens the infection process and further promotes the spread of the disease. The p53 expression is modulated following HIV-1 infection (Cooper et al., 2013b; Cooper et al., 2013a). It is increased by miR-124a overexpression (Shi et al., 2013), whereas miR-34a and let-7c are known targets of the p53 protein (Tarasov et al., 2007; Boominathan, 2010). This might be a possible molecular pathway exploited by the virus in order to inhibit TASK1 and p21 (Fig. 8). In the future, it would be of interest to further investigate whether the p53 pathway is, in fact, being regulated by the virus in order to overpower the two important proteins in the innate inhibition mechanisms – p21 and TASK1.

In conclusion, we observed a direct correlation between three cellular miRNAs and the ability of the virus to replicate and spread. HIV-1 manipulates let-7c, miR-34a and miR-124a miRNA expression in order to downregulate their targets, p21 and TASK1 restriction factors. Counteracting this link between miRNAs and restriction factor target genes might lead to the development of novel therapeutic strategies. Inhibition of miRNAs that target or modulate cellular antiviral proteins and restriction factors might enhance the antiviral responses triggered by HIV-1 entry and infection, which can potentially result in an increased resistance to productive infection and decrease the susceptibility of the infected cell to productive HIV-1 infection.

## MATERIALS AND METHODS

### Cell culture

T-lymphocytes (Jurkat, H9, Sup-T1, T1 and JLTRG-R5) were grown in standard RPMI medium, whereas HeLa-CCR5 and HEK293 were grown in standard DMEM. MAGI cells are HeLa origin cells, which express high levels of CD4 receptor and contain a single integrated copy of a β-galactosidase gene under the control of a truncated HIV-1 LTR (Kimpton and Emerman, 1992). MAGI-CCR5 expresses the human chemokine receptor, CCR5 (Deng et al., 1996). JLTRG-R5 cells, derived from Jurkat human T-cells, have been stably transfected with an LTR-GFP construct (Kutsch et al., 2004).

### Virus preparation

HEK293 cells were transfected with HIV-1 (pSVC21) wild-type plasmid using TurboFect cell transfection reagent (Fermentas, Lithuania) according to the manufacturer's instructions.

### Viruses and cell line infections

Full-length HIV-1_HXB2_ strain was generated from the pSVC21 plasmid, as described previously (Hayouka et al., 2007). For T1 lymphocytes infection, two million cells were infected with two million virions of HIV-1, thus multiplicity of infection (MOI) = 1. For Sup-T1 and H9 lymphocytes infection, two million SupT1 or H9 lymphocyte cells were infected with 40,000 virions of HIV-1 (MOI = 0.2). At 4 days later, two million fresh cells were added to the culture. At 8 days post infection, cells were harvested and total RNA was extracted using Trizol. For HeLa-CCR5 infection, 24 hours post miRNA overexpression or inhibition, 1×10^4^ HeLa-CCR5 cells per well were plated in a 96-well plate. A day later, the cells were infected with MOI = 0.01. For JLTRG-R5 infection, 48 hours post miRNA overexpression or inhibition, 0.3×10^6^ JLTRG-R5 cells per well were spread in black 96-well plates (Greiner, Germany) and infected with MOI = 0.01. At 48 hours or 5 days post HeLa-CCR5 or JLTRG-R5 viral infection, supernatant samples were collected and viral expression was analyzed by RT-qPCR. Wild-type Moloney strain MLV (MMLV, 5µl) were added to the supernatant and served as the loading control for real-time PCR (Voronin et al., 2014). Production of GFP by HIV-1-infected cells was measured daily for 14 or 21 consecutive days in a Synergy^TM^ HT Multi-Detection Microplate Reader (Bio-Tek Instruments, USA), equipped with the following filter set: excitation, 485/20 nm; emission, 528/20 nm. The results of the control empty miRVec or scrambled RNA sequence infections were deducted for normalization.

### RNA extraction and miRNA profiling

Total RNA was extracted from cell cultures using Trizol (Bio-Lab Ltd, Israel) or miRNeasy Mini Kit (Qiagen, Germany). The final RNA concentration and purity were measured using a NanoDrop ND-1000 spectrophotometer (NanoDrop Technologies, Thermo Scientific, USA).

The TLDAs are quantitative real-time (RT)-PCR assays (Life Technologies, USA). cDNA samples were loaded on Human TLDA card A, according to the manufacturer's instructions. PCR amplification was performed using ABI Prism 7900HT Sequence Detection System. The results were analyzed with SDS software (Applied Biosystems). The Ct for each miRNA and endogenous control U6 in each sample were used to create ΔCt values (Ct_miRNA_–Ct_U6_). Thereafter, ΔΔCt values were calculated by subtracting the ΔCt of the non-infected Sup-T1 cells from the Ct value of HIV-1-infected Sup-T1 cells.

### Deep sequencing and analysis

Samples for deep sequencing analysis were prepared from 10 µg of each sample following Illumina's Small RNA sample preparation protocol (v1.5). During this process, samples were ligated with 3′ and 5′ adaptors, reverse transcribed and then amplified using PCR. Illumina Genome Analyzer IIx instrument (USA) was used for sequencing.

The data sequences were screened for the sequence of the small-RNA adaptor, and the adaptor sequences were trimmed using standard settings in Illumina's GAPipeline1.0. Processed Illumina data was managed by RandA software (Isakov et al., 2012). The reads were aligned to the human subset miRNAs in the miRbase database using BWA-aligner software. The number of reads was standardized by mapping each transcript according to its length and the initial total number of mapped reads in the sample based on the ‘reads per kilo-base per million’ (RPKM) method (Mortazavi et al., 2008). Only perfect matches were counted in the main analysis. Next, results were ranked in terms of differentially expressed miRNAs between the two samples. Statistical analysis was performed using a chi-square distribution.

### miRNA constructs

Pre-miRNAs were cloned into the *Bam*HI*–EcoRI* restriction site of the miRNA expression vector miRVec, which was provided by Prof. Reuven Agami (Voorhoeve et al., 2006). The genomic loci of ∼70 bp upstream and downstream of the pre-miRNAs were inserted into the vector.

### miRNA transfections and inhibitors

T-cells were seeded in 12-well plates at a concentration of 1.6×10^6^ cells/well, whereas HeLa-CCR5 cells were seeded in six-well plates at a concentration of 2×10^5^ cells/well. Transfections were performed using Lipofectamine 2000 transfection reagent (Invitrogen, USA) according to the manufacturer's instructions. Transfection efficiencies were measured by GFP fluorescence in all cells, indicating a transfection efficiency of at least 50% repeatedly. For miRNA overexpression, transfection was performed in T-cells with 2 µg and in HeLa-CCR5 with 1.25 µg of miRVec containing the desired pre-miRNA or an empty vector. For miRNA inhibition, 15 ng AntagoMiR (miRNA Inhibitors) or a scrambled inhibitor (Ambion Applied Biosystems, USA) were used.

### Real-time PCR

Reverse transcription reactions for mRNA and for specific mature miRNAs were performed using random primer and High-Capacity cDNA Reverse-Transcription Kit or TaqMan miRNA Assays, respectively, according to manufacturer's protocol (Life Technologies, USA). The expression of single miRNAs or mRNAs was tested similarly using TaqMan Universal PCR Master Mix (Life Technologies, USA) or SYBR green PCR master mix (Life Technologies, USA), respectively. The PCR amplification and reading was performed using the Step-One Detection System. The endogenous controls U6 and RNU6B were not stably expressed, and were therefore replaced by miR-181a. mRNA expression levels were normalized to those of GAPDH.

### Dual luciferase assay

Fragments of ∼300–350 bp of CDKN1A and TASK1 3′UTR spanning the miRNA-binding sites were cloned downstream of the *Renilla* luciferase reporter of the psiCHECK^TM^-2 plasmid (Promega, USA), which also contains a firefly luciferase reporter (used as control). The miRNA-binding sites were mutated by using QuikChange Lightning Site-Directed Mutagenesis Kit (Agilent, USA).

For luciferase assays, HeLa-CCR5 cells were transfected using Lipofectamine 2000 transfection reagent with 5 ng of psiCHECK-2 plasmid containing the desired 3′UTR with or without site-directed mutations and 485 ng miRVec containing the desired pre-miRNA or an empty vector. At 48 hours after transfection, firefly and *Renilla* luciferase activities were measured using the Dual Luciferase reporter assay system kit (Promega, USA) and LUMIstar Omega Luminometer (BMG LabTech, Germany), according to Promega's instructions.

### Western blot analysis

H9 cell lines were homogenized with lysis buffer. Protein levels in the lysates were determined by using the Bio-Rad protein assay (Bio-Rad, USA). Lysates were resolved by SDS-PAGE through 4–12% gels (GeBaGel, Belgium) and transferred by electroporation to nitrocellulose membranes. Membranes were blotted with anti-p21 (Santa Cruz Biotechnology, USA) or anti-actin (Millipore, USA) antibodies, followed by a secondary antibody linked to horseradish peroxidase. Band quantification was performed using ImageJ software (National Institutes of Health, USA).

### Intracellular staining of p21

At 72 hours post Jurkat transfection with let-7c or empty miRVec, cells were fixed with 4% paraformaldehyde (EMS, USA) and permeabilized with 0.1% saponin (Sigma-Aldrich, USA). Next, cells were incubated with rabbit anti-p21 antibody conjugated to Alexa Fluor 647 (Cell Signaling Technology, USA). Fluorescent signal was detected by using a Gallios FACS machine (Beckman Coulter, USA). Rabbit IgG antibody conjugated to Alexa Fluor 647 (Cell Signaling Technology, USA) served as an isotype control. The p21 expression was determined by using Flowing software 2 (Cell Imaging Core, Turku Centre for Biotechnology, Finland).

### Cell cycle and proliferation analysis

Both parameters were measured using FITC BrdU Flow Kit (BD Biosciences, USA) according to the manufacturer's protocol. At 4 days following Jurkat transfection with let-7c or empty miRVec, cells were incubated with bromodeoxyuridine (BrdU) for 30 minutes. BrdU and 7-AAD staining was detected by using the Gallios FACS machine and was analyzed using Flowing software 2.

### The multinucleate activation of galactosidase indicator infection assay

1×10^4^ MAGI-CCR5 cells transfected with let-7c antisense and 1×10^3^ MAGI-CCR5 cells transfected with miR-34a or miR-124a antisense were plated in a 96-well plate at 24 hours following antisense overexpression. At 24 hours later, cells were infected with HIV-1 with MOI = 0.01. At 48 hours post infection for let-7c antisense overexpression, or five days post infection for miR-34a or miR-124a antisense overexpression, cells were fixed with 1% formaldehyde and stained with X-gal staining solution for 1 hour. Positive infections were counted under light microscopy in triplicate (Rosenbluh et al., 2007).

### Statistical analysis

Data are presented as the mean±s.e.m. *P*-values were calculated using a paired Student's *t*-test and chi-square distribution, with *P*<0.05 considered as significant for both statistical tests.

## Supplementary Material

Supplementary Material
