# Supplementary material for: MicroRNA-mediated regulation of p21 and TASK1 cellular restriction factors enhances HIV-1 infection
Source: J Cell Sci. 2015 Apr 15;128(8):1607–16. doi: 10.1242/jcs.167817 (PMC4406127; doi:10.1242/jcs.167817)
Supplement: Supplementary Material [file supp_128_8_1607__index.html]

Supplementary Material 

# MicroRNA‐mediated regulation of p21 and TASK1 cellular restriction factors enhances HIV‐1 infection

## JCS167817 Supplementary Material

**Files in this Data Supplement:**

- **Supplementary Material**
