# Supplementary material for: MicroRNA-mediated regulation of p21 and TASK1 cellular restriction factors enhances HIV-1 infection
Source: J Cell Sci. 2015 Apr 15;128(8):1607–16. doi: 10.1242/jcs.167817 (PMC4406127; doi:10.1242/jcs.167817)
Supplement: Supplementary Material [file supp_128.8.1607_JCS167817.pdf]

MicroRNA regulation of p21 and TASK1 cellular restriction-factors enhances HIV-1 infection

Luba Farberov[1], Eytan Herzig[1], Shira Modai[1], Ofer Isakov[1], Amnon Hizi[1], Noam Shomron[1*]

[1]Faculty of Medicine, Tel Aviv University, Tel Aviv 69978, Israel

* Corresponding author: N Shomron, TEL +972-3-640-6594, nshomron@post.tau.ac.il

Table S1. Differently expressed miRNAs by more than 2 FC detected using the deep sequencing method in Sup-T1 or T1 lymphocytes cell-lines . (A) 126 miRNAs which were up-regulated by more than 2 FC in the Sup-T1 cell-line. (B) 25 miRNAs which were down-regulated by more than 2 FC in the Sup-T1 cell-line. (C) 36 miRNAs which were up-regulated by more than 2 FC in the T1 cell-line. (D) 54 miRNAs which were down-regulated by more than 2 FC in the T1 cell-line.

Table S2. Differently expressed miRNAs by more than 2 FC detected using the TaqMan low-density array method in Sup-T1 lymphocyte cell-line. (A) 72 miRNAs which were up-regulated by more than 2 FC in the Sup-T1 cell-line. (B) 60 miRNAs which were down-regulated by more than 2 FC in the Sup-T1 cell-line.

Figure S1. Expression of let-7c, miR-34a and miR-124a following HIV-2 infection for 24 hours, 48 hours and 5 days. Normalized qPCR fold change results are presented.

Figure S2. The p21 protein is directly regulated by let-7c. Western blot analysis of p21 and actin in H9 cell-line following 72 hours of let-7c OE, (A) sample gel and (B) quantization of three experiments. Bands quantification was done using ImageJ software. ($n=3$, $*p<0.05$.)

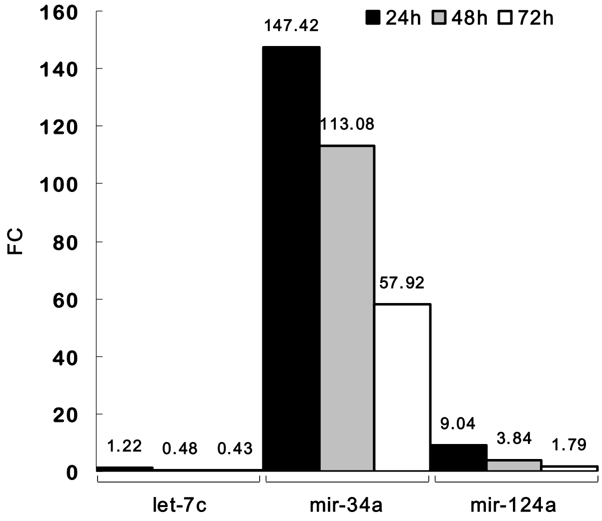
160
140
120
100
80
60
40
20
0
FC
24h
48h
72h
147.42
113.08
57.92
1.22
0.48
0.43
9.04
3.84
1.79
let-7c
mir-34a
mir-124a

Figure S1

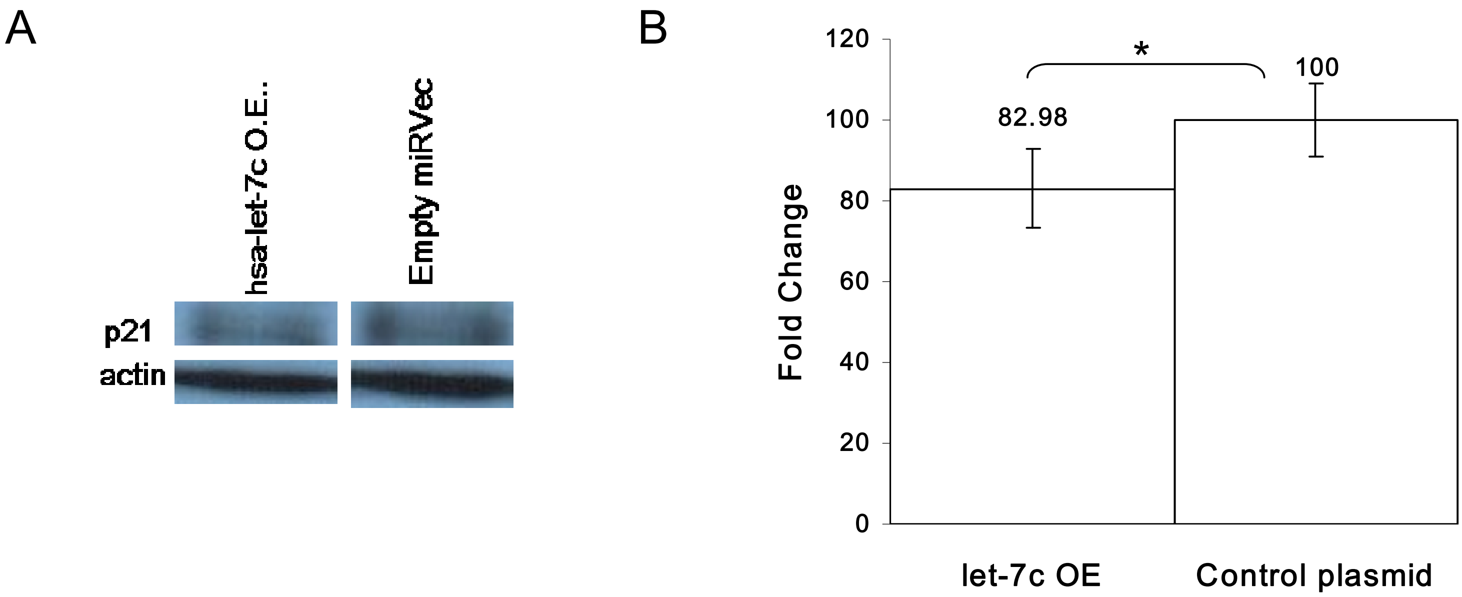
A
hsa-let-7c O.E..
Empty miRVec
p21
actin
B
120
100
80
60
40
20
0
Fold Change
*
82.98
100
let-7c OE
Control plasmid

Figure S2

MicroRNA regulation of p21 and TASK1 cellular restriction-factors enhances HIV-1 infection

Luba Farberov[1], Eytan Herzig[1], Shira Modai[1], Ofer Isakov[1], Amnon Hizi[1], Noam Shomron[1*]

[1]Faculty of Medicine, Tel Aviv University, Tel Aviv 69978, Israel

* Corresponding author: N Shomron, TEL +972-3-640-6594, nshomron@post.tau.ac.il

(A)

FC	miRNA	FC	miRNA	FC	miRNA
2.895	hsa-miR-3184	7.128	hsa-miR-1291	998.440	hsa-miR-1290
2.851	hsa-miR-3647-5p	7.128	hsa-miR-3651	331.039	hsa-miR-1246
2.851	hsa-let-7g*	7.128	hsa-miR-1257	263.732	hsa-miR-608
2.851	hsa-let-7a*	7.128	hsa-miR-1295	114.046	hsa-miR-1203
2.645	hsa-miR-324-5p	7.128	hsa-miR-3192	57.023	hsa-miR-3687
2.640	hsa-miR-331-5p	7.128	hsa-miR-662	36.443	hsa-miR-184
2.626	hsa-miR-3909	7.128	hsa-miR-139-5p	33.755	hsa-miR-937
2.376	hsa-miR-1271	7.128	hsa-miR-877*	28.512	hsa-miR-1288
2.376	hsa-miR-628-5p	6.480	hsa-miR-874	28.512	hsa-miR-762
2.376	hsa-miR-1226	6.178	hsa-miR-146a	28.512	hsa-miR-1972
2.376	hsa-let-7i*	5.702	hsa-miR-203	21.384	hsa-miR-4301
2.376	hsa-miR-30b*	5.702	hsa-miR-1273d	21.384	hsa-miR-3913
2.376	hsa-miR-3607-3p	5.578	hsa-miR-629	21.384	hsa-miR-3141
2.376	hsa-miR-3157	5.346	hsa-miR-3127	21.384	hsa-miR-1234
2.376	hsa-miR-3121	5.252	hsa-miR-375	17.820	hsa-miR-760
2.376	hsa-miR-19a*	5.106	hsa-miR-192	16.038	hsa-miR-574-5p
2.376	hsa-miR-580	5.094	hsa-miR-215	14.256	hsa-miR-10a
2.376	hsa-miR-296-5p	4.752	hsa-miR-3688	14.256	hsa-miR-10b
2.376	hsa-miR-193a-3p	4.752	hsa-miR-3615	14.256	hsa-miR-3187
2.376	hsa-miR-20a*	4.752	hsa-miR-1284	14.256	hsa-miR-3664
2.257	hsa-miR-1296	4.455	hsa-miR-3065-3p	14.256	hsa-miR-615-3p
2.251	hsa-miR-138-1*	4.424	hsa-miR-425*	14.256	hsa-miR-1538

2.248	hsa-miR-877	4.386	hsa-miR-570	14.256	hsa-miR-1224-5p
2.247	hsa-miR-150	4.281	hsa-miR-152	14.256	hsa-miR-4298
2.197	hsa-miR-625	4.277	hsa-miR-193a-5p	14.256	hsa-miR-297
2.193	hsa-miR-1180	4.269	hsa-miR-23a	11.880	hsa-miR-196a
2.187	hsa-miR-130b*	3.734	hsa-miR-143	11.405	hsa-miR-205
2.139	hsa-miR-30e	3.620	hsa-miR-210	10.692	hsa-miR-1236
2.138	hsa-miR-135a	3.564	hsa-miR-3065-5p	9.942	hsa-miR-3613-5p
2.131	hsa-miR-502-3p	3.564	hsa-miR-33b	8.910	hsa-miR-1307
2.112	hsa-miR-93*	3.564	hsa-miR-22*	7.128	hsa-miR-491-3p
2.096	hsa-miR-671-5p	3.564	hsa-miR-29a*	7.128	hsa-miR-1
2.083	hsa-miR-26b	3.564	hsa-miR-3647-3p	7.128	hsa-miR-193b*
2.056	hsa-miR-361-5p	3.562	hsa-miR-30d*	7.128	hsa-miR-665
2.050	hsa-miR-129-3p	3.445	hsa-miR-642a	7.128	hsa-miR-1266
2.037	hsa-miR-3912	3.288	hsa-miR-500a*	7.128	hsa-miR-3123
2.035	hsa-miR-374a	3.168	hsa-miR-3138	7.128	hsa-miR-2110
2.020	hsa-miR-378*	3.162	hsa-miR-23b	7.128	hsa-miR-1179
		3.009	hsa-miR-128	7.128	hsa-miR-3907
		2.970	hsa-miR-1303	7.128	hsa-miR-1248
		2.945	hsa-miR-374b	7.128	hsa-miR-1282
		2.939	hsa-miR-652	7.128	hsa-miR-548p
		2.916	hsa-miR-454*	7.128	hsa-miR-3692*
		2.916	hsa-miR-660	7.128	hsa-miR-3620

126 miRNAs which were up-regulated by more than 2 FC in the Sup-T1 cell-line.

(B)

FC	miRNA	FC	miRNA	FC	miRNA
0.324	hsa-miR-3177	0.467	hsa-miR-33a*	0.494	hsa-miR-3928
0.285	hsa-miR-191*	0.445	hsa-miR-16-1*	0.482	hsa-miR-17*
0.228	hsa-miR-423-5p	0.445	hsa-miR-301a	0.482	hsa-miR-342-3p
0.223	hsa-miR-590-5p	0.401	hsa-miR-181a*	0.481	hsa-let-7c
0.216	hsa-miR-27a*	0.396	hsa-miR-424*	0.480	hsa-miR-130b
0.192	hsa-miR-1260	0.388	hsa-miR-29c	0.479	hsa-miR-365
0.170	hsa-miR-3613-3p	0.375	hsa-miR-219-1-3p	0.473	hsa-let-7d
0.069	hsa-miR-92a-1*	0.344	hsa-miR-191	0.471	hsa-miR-106b
0.061	hsa-miR-106a				

25 miRNAs which were down-regulated by more than 2 FC in the Sup-T1 cell-line.

T1

(C)

FC	miRNA	FC	miRNA	FC	miRNA
2.285	hsa-miR-320b	2.748	hsa-miR-660	4.529	hsa-miR-1290
2.281	hsa-miR-100	2.707	hsa-miR-1	4.264	hsa-miR-135a
2.275	hsa-miR-877*	2.690	hsa-miR-548j	4.113	hsa-miR-146a*
2.244	hsa-miR-328	2.631	hsa-miR-125a-5p	3.344	hsa-miR-3676
2.199	hsa-miR-335*	2.576	hsa-miR-361-3p	3.344	hsa-miR-629
2.185	hsa-let-7i*	2.568	hsa-miR-582-5p	3.310	hsa-miR-1246
2.162	hsa-miR-148a	2.563	hsa-miR-330-5p	3.194	hsa-let-7b
2.123	hsa-miR-1266	2.548	hsa-miR-151-3p	2.986	hsa-miR-616*
2.123	hsa-miR-1254	2.395	hsa-miR-99b	2.945	hsa-miR-582-3p
2.070	hsa-miR-151-5p	2.388	hsa-miR-500a	2.911	hsa-miR-130a
2.047	hsa-miR-550a	2.388	hsa-miR-1271	2.831	hsa-miR-629*
2.027	hsa-miR-138-1*	2.315	hsa-miR-320c	2.787	hsa-miR-513b

36 miRNAs which were up-regulated by more than 2 FC in the T1 cell-line.

(D)

FC	miRNA	FC	miRNA	FC	miRNA
0.299	hsa-miR-1278	0.409	hsa-miR-3613-3p	0.500	hsa-miR-3942
0.287	hsa-miR-615-5p	0.398	hsa-miR-185*	0.478	hsa-miR-3692
0.259	hsa-miR-558	0.393	hsa-miR-150	0.478	hsa-miR-3682
0.259	hsa-miR-3681	0.392	hsa-miR-3131	0.477	hsa-miR-1274a
0.250	hsa-miR-548w	0.392	hsa-miR-99b*	0.472	hsa-miR-3158
0.220	hsa-miR-223	0.387	hsa-miR-23a*	0.472	hsa-miR-3913
0.199	hsa-miR-3123	0.367	hsa-miR-1304	0.468	hsa-miR-2277-5p
0.190	hsa-miR-1261	0.363	hsa-miR-548u	0.464	hsa-miR-3149
0.172	hsa-miR-1276	0.359	hsa-miR-3678-3p	0.451	hsa-miR-222
0.168	hsa-miR-671-5p	0.359	hsa-miR-3168	0.448	hsa-miR-181a*
0.160	hsa-miR-1273e	0.347	hsa-miR-1305	0.447	hsa-miR-720
0.159	hsa-miR-2276	0.336	hsa-miR-34c-5p	0.443	hsa-miR-27a*
0.148	hsa-miR-580	0.332	hsa-miR-3142	0.440	hsa-miR-625*
0.113	hsa-miR-1275	0.329	hsa-miR-183*	0.431	hsa-miR-145
0.092	hsa-miR-641	0.325	hsa-miR-1280	0.427	hsa-miR-424*
0.089	hsa-miR-503	0.318	hsa-miR-3164	0.425	hsa-miR-3127
0.074	hsa-miR-656	0.318	hsa-miR-221	0.420	hsa-miR-374a*
0.068	hsa-miR-3681*	0.301	hsa-miR-548n	0.419	hsa-miR-548k

54 miRNAs which were down-regulated by more than 2 FC in the T1 cell-line.

MicroRNA regulation of p21 and TASK1 cellular restriction-factors enhances HIV-1 infection

Luba Farberov[1], Eytan Herzig[1], Shira Modai[1], Ofer Isakov[1], Amnon Hizi[1], Noam Shomron[1*]
[1]Faculty of Medicine, Tel Aviv University, Tel Aviv 69978, Israel
* Corresponding author: N Shomron, TEL +972-3-640-6594, nshomron@post.tau.ac.il

(A)

FC	miRNA	FC	miRNA	FC	miRNA
352843.53	hsa-miR-10a	206.02	hsa-miR-199a-3p	186.44	hsa-miR-367
3.67	hsa-miR-126	2.66	hsa-miR-200c	2519.84	hsa-miR-376a
29.36	hsa-miR-133a	36.72	hsa-miR-202	14.99	hsa-miR-381
2.10	hsa-miR-138	3.46	hsa-miR-210	16284.45	hsa-miR-382
2.01	hsa-miR-139-5p	15212.44	hsa-miR-211	271.34	hsa-miR-410
2027.08	hsa-miR-146a	879610.75	hsa-miR-212	2.40	hsa-miR-454
3.41	hsa-miR-146b-5p	3.24	hsa-miR-215	8.33	hsa-miR-483-5p
33992.68	hsa-miR-147	2.13	hsa-miR-222	2.59	hsa-miR-485-3p
2.65	hsa-miR-148b	1238.50	hsa-miR-23a	1822.62	hsa-miR-486-5p
2.18	hsa-miR-16	518408.66	hsa-miR-27b	93405.45	hsa-miR-487b
3.31	hsa-miR-183	2.80	hsa-miR-29a	4773.19	hsa-miR-490-3p
40.22	hsa-miR-184	15.46	hsa-miR-31	5.59	hsa-miR-494
2.42	hsa-miR-185	958.24	hsa-miR-323-3p	22.18	hsa-miR-502-3p
2.32	hsa-miR-186	2.33	hsa-miR-324-3p	154726.90	hsa-miR-509-5p
2.03	hsa-miR-192	2.27	hsa-miR-330-3p	1551.36	hsa-miR-511
112.21	hsa-miR-193a-3p	2.31	hsa-miR-337-5p	5789.74	hsa-miR-515-5p
2.07	hsa-miR-361-5p	2.64	hsa-miR-345	86.59	hsa-miR-517b
25.02	hsa-miR-518b	89.68	hsa-miR-520a-3p	119.09	hsa-miR-548c-5p
50432.25	hsa-miR-518e	351.03	hsa-miR-523	2.08	hsa-miR-574-3p
72.02	hsa-miR-518f	161692.22	hsa-miR-542-5p	20943.98	hsa-miR-579
11566.99	hsa-miR-519a	4.05	hsa-miR-545	1000.34	hsa-miR-582-3p
49.56	hsa-miR-519d	176163.05	hsa-miR-548b-3p	3.78	hsa-miR-590-5p

10.54	hsa-miR-616	2.59	hsa-miR-628-5p	31.70	hsa-miR-672
59.71	hsa-miR-618	2.63	hsa-miR-660	93.08	hsa-miR-708

72 miRNAs which were up-regulated by more than 2 FC in the Sup-T1 cell-line.

(B)

FC	miRNA	FC	miRNA	FC	miRNA
2.19E-05	hsa-miR-423-5p	7.23E-04	hsa-miR-362-3p	0.166	hsa-miR-671-3p
3.61E-05	hsa-miR-152	7.90E-04	hsa-miR-429	0.204	hsa-miR-136
4.23E-05	hsa-miR-375	0.001	hsa-miR-874	0.265	hsa-miR-302c
4.57E-05	hsa-miR-145	0.001	hsa-let-7b	0.280	hsa-miR-142-5p
8.36E-05	hsa-miR-100	0.001	hsa-miR-339-5p	0.340	hsa-miR-221
9.22E-05	hsa-miR-125b	0.001	hsa-miR-582-5p	0.344	hsa-let-7a
9.38E-05	hsa-miR-193a-5p	0.001	hsa-let-7c	0.362	hsa-miR-27a
1.21E-04	hsa-miR-501-3p	0.001	hsa-miR-187	0.398	hsa-miR-342-5p
1.68E-04	hsa-miR-548d-3p	0.002	hsa-miR-125a-3p	0.404	hsa-miR-129-3p
1.93E-04	hsa-miR-101	0.003	has-miR-155	0.418	hsa-miR-92a
2.01E-04	hsa-miR-502-5p	0.003	hsa-miR-129-5p	0.433	hsa-miR-301a
2.48E-04	hsa-miR-32	0.004	hsa-miR-505	0.443	hsa-miR-29c
2.83E-04	hsa-miR-450b-5p	0.004	hsa-miR-503	0.452	hsa-miR-515-3p
3.02E-04	hsa-miR-23b	0.004	hsa-miR-576-5p	0.458	hsa-miR-197
3.77E-04	hsa-miR-629	0.008	hsa-miR-518d-3p	0.479	hsa-miR-93
4.19E-04	hsa-miR-219-1-3p	0.018	hsa-miR-489	0.067	hsa-miR-376c
5.14E-04	hsa-miR-139-3p	0.020	hsa-miR-200a	0.102	hsa-miR-487a
5.39E-04	hsa-miR-570	0.032	hsa-miR-362-5p	0.109	hsa-miR-130a
5.39E-04	hsa-miR-548d-5p	0.050	hsa-miR-99a	0.159	hsa-miR-99b
6.44E-04	hsa-miR-597	0.001	hsa-miR-135a	0.001	hsa-miR-340

60 miRNAs which were down-regulated by more than 2 FC in the Sup-T1 cell-line.
